# Weather Extremes in the Mediterranean Winter Are Associated With Reduced Apparent Survival and Delayed Initiation of Egg‐Laying in a Migratory Raptor

**DOI:** 10.1002/ece3.72741

**Published:** 2025-12-18

**Authors:** Inga Kujala, Carina Nebel, Hannu Pöysä, Erkki Korpimäki

**Affiliations:** ^1^ Department of Environmental and Biological Sciences University of Eastern Finland Joensuu Finland; ^2^ Section of Ecology, Department of Biology University of Turku Turku Finland; ^3^ Turku Collegium for Science, Medicine and Technology University of Turku Turku Finland

**Keywords:** apparent survival, breeding phenology, carry‐over effects, Eurasian kestrel, weather effects

## Abstract

Climate change increases the occurrence of extreme weather conditions, affecting ecosystems worldwide but possible impacts of these extremes on biological systems have been insufficiently studied. Among others, migratory raptors are particularly susceptible to adverse weather conditions. We analysed capture‐recapture data of Eurasian Kestrels ( *Falco tinnunculus* ) breeding in western Finland during 1985–2018 to study how environmental conditions in their boreal breeding areas and Mediterranean wintering areas affect adult survival rate. As predictors of apparent survival, we used density data of their primary prey (voles) from their breeding grounds, alongside seasonal minimum temperatures and rainy‐day frequencies of Finnish summer and Mediterranean winter. We also tested whether adverse weather in winter can result in carry‐over delays in the initiation of next breeding season (i.e., egg‐laying). We found that both frequent and infrequent rain in winter predicted low apparent survival rate, while summer weather conditions had no effect on survival. Vole abundance in breeding areas negatively correlated with apparent survival. Finally, frequent precipitation in Mediterranean wintering grounds resulted in delayed laying in the following spring during years of low vole abundance in the breeding grounds. We conclude that weather extremes in wintering areas are more acutely affecting annual adult survival in long‐distance migratory kestrels than weather conditions in breeding grounds, thus providing the first evidence of impacts of climate extremes on survival rate of migratory species in North Europe. Furthermore, our results suggest that effects of such disadvantageous weather conditions carry over from winter to spring. If climate change amplifies the frequency and magnitude of weather extremes in the Mediterranean, its long‐distance migrant inhabitants may encounter setbacks in population growth and stability.

## Graphical abstract

We analysed capture‐recapture data of breeding Eurasian kestrels ( *Falco tinnunculus* ) in western Finland during 1985–2018 to study how environmental conditions in their boreal breeding areas and Mediterranean wintering areas affect adult survival rate and timing of laying. We found that both frequent and infrequent precipitation in winter predicted reduced apparent survival rate and that frequent precipitation in wintering grounds resulted in delayed laying during years of poor prey availability.

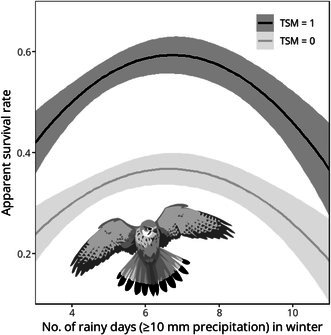

## 1 Introduction

Globally, human‐induced climate change is a pressing threat to biodiversity (Jaureguiberry et al. 2022). Aside from increasing mean temperatures, weather anomalies such as extreme precipitation and droughts are predicted to occur more frequently, but with climate trends varying spatially (IPCC 2023). Ecological consequences of extreme weather conditions for wildlife have only recently received attention (Bailey and van de Pol 2016; Murali et al. 2023; Preston‐Allen et al. 2024), with migratory species being in a uniquely vulnerable position. Migratory species rely on the suitability of several habitats, including breeding and wintering grounds as well as stopover sites (Runge et al. 2015). Furthermore, migratory species encounter an increased risk of phenological mismatches, resulting from asynchronous responses to climate change across their seasonal habitats and migration networks (Both et al. 2010; Wei et al. 2024). Climate‐related threats to migrants can go unnoticed if their population parameters and demographic traits are studied in the context of their breeding habitats alone.

Migration can be costly, as mortality tends to peak during migration (Newton 2007; Klaassen et al. 2014; Rockwell et al. 2017). As migration is energetically demanding, migrants are particularly sensitive to environmental conditions (Brlík et al. 2022). Additionally, the consequences of disadvantageous weather in non‐breeding habitats can also carry over to the breeding season and reduce reproductive performance (Saino et al. 2004; Harrison et al. 2011; Swift et al. 2020). Hence, to fully comprehend how vital rates are affected by climate change, relevant climate conditions should be considered for each phase of the annual cycle.

Adult survival rate is a key demographic trait affecting avian populations (Sæther and Bakke 2000). In the Palearctic‐African migratory system, survival studies concerning the weather in the wintering grounds of migrants have been mostly conducted on insectivorous birds wintering in sub‐Saharan regions (e.g., Saino et al. 2004; Boano et al. 2020), but few studies (e.g., Mihoub et al. 2010; Sergio et al. 2022) address the influence of weather conditions on the survival of migrant raptors or concern raptors wintering around the Mediterranean Sea. Based on our long‐term monitoring of migratory boreal Eurasian Kestrels ( *Falco tinnunculus* ), we attempt to shed new light on how climate change impacts adult survival in migratory raptors.

Besides directly influencing population demography by affecting mortality, overwinter conditions can result in carryover effects reflected in breeding success and timing, both of which affect the eventual recruit production (Dunn and Winkler 2010; O'Connor et al. 2014; Shipley et al. 2020). Environmental stress experienced during the non‐breeding season can delay the onset of spring migration, the initiation of egg‐laying or both (Descamps et al. 2011; Tøttrup et al. 2012). Hence, the effects of extreme environmental conditions are expected to cumulate upon different demographic parameters, even if they occur outside the reproductive season.

The Eurasian Kestrel (hereafter ‘kestrel’) is a diurnal raptor with widespread distribution across the Eurasian and African continents (Village 1990). In Europe, southern populations are sedentary, while partially migrant populations occur in Central Europe and full migrants in North Europe (Village 1990; Holte et al. 2016). Kestrels breeding in North Europe spend their winters between western continental Europe and sub‐Saharan Africa, with the northernmost populations migrating furthest south (Wallin et al. 1987; Village 1990; Saurola et al. 2013). Kestrels in North Europe also display varying levels of long‐distance dispersal, which are non‐seasonal and non‐directional movements, with adults and males showing higher site fidelity than juveniles and females (Vasko et al. 2011; Terraube et al. 2015). At the North European breeding grounds, kestrels primarily feed on voles of the genera *Microtus* and *Myodes* (Korpimäki 1985; Village 1990) while in the Mediterranean, kestrel diet frequently includes lizards, passerines and Coleoptera and Orthoptera insects (Carrillo et al. 2017; Fargallo et al. 2020). The abundance of most prey is dependent on vegetation availability, which can be limited by drought in the semi‐arid Mediterranean kestrel habitats (Gouveia et al. 2016; Chidodo et al. 2020; Klein et al. 2020). Moreover, as open‐area predators, the foraging efficiency of kestrels is highly affected by weather conditions; precipitation, in particular, can reduce hunting success (Rijnsdorp et al. 1981; Village 1990).

While the global kestrel population has remained large, numbers in Central and South Europe as well as in African savanna are decreasing (BirdLife International 2024; Shaw et al. 2024). Concerning population declines are occurring in the British Isles; for example, during the 27‐year survey interval in Great Britain, the kestrel population was estimated to have decreased by 40% (Gilbert et al. 2021; Heywood et al. 2024). These declines reflect the global trend of population losses among raptors (McClure et al. 2018). Within its broad distribution range, kestrels comprise numerous widely monitored populations of different migratory strategies. Due to this, they are an ideal model species for disentangling various environmental processes affecting population fluctuations in raptors.

Here, we examined how different environmental and individual variables are associated with annual rates of apparent adult survival (which is the combination of mortality and emigration, hereafter ‘survival’) of breeding individuals in a boreal population of migratory kestrels. We use 29 years of mark‐recapture data to (1) examine the relationship between survival and climate change‐sensitive weather conditions in the wintering areas. These variables include the number of days with precipitation ≥ 10 mm (hereafter ‘R10’) and the seasonal minimum temperature (formally the minimum value of daily minimum temperature; hereafter ‘TNn’) in winter. As drought limits food availability while foraging success is reduced by frequent rain, we predict that both excessive and scarce rainfall are disadvantageous regarding the survival of kestrels, and that mild winters, represented by high TNn, are associated with elevated survival compared to winters with more extreme cold spells. Moreover, (2) we tested whether corresponding weather variables in breeding areas affected survival, predicting a negative relationship between survival and both the R10 and TNn of summer. This prediction is based on the prior observations of adverse weather conditions adding to the costs of reproduction and thus having potential negative effects on breeding individuals (Zabala et al. 2022). Also, (3) we examined the relationship between food availability in the early breeding season (measured as an abundance index of voles) and survival. As springtime food availability is strongly associated with reproductive effort and success of kestrels (Tolonen and Korpimäki 1994; Korpimäki and Wiehn 1998) and the availability of main foods throughout the breeding season, we expected vole abundance to influence survival of adult kestrels. We predicted a positive association between survival and food availability, indicative of the ease of foraging and maintaining body condition reflected as increased survival (Hakkarainen et al. 2002; Oro et al. 2021; Korpimäki et al. 2025). Additionally, (4) we examined whether different age groups diverge in their survival rates or whether their survival is affected differently by precipitation in either wintering or breeding grounds, as age‐specific responses have been documented in other raptors (Mihoub et al. 2010; Zabala et al. 2022). Lastly, (5) we tested whether the weather conditions in wintering grounds influence the initiation date of egg‐laying (hereafter ‘laying date’). We included weather effects (R10, TNn) as explanatory variables both independently and interactively with food availability, which is a known predictor of laying date (Korpimäki and Wiehn 1998). By this, we aimed to determine if the adverse wintering conditions can have long‐term consequences on the individual level, carrying over to breeding season in addition to influencing survival.

## 2 Materials and Methods

### 2.1 Study Area and Data Collection

Our study population of kestrels is located in the area of Kauhava and Lapua (western Finland, approx. 63° N, 23° E). The landscape of the 1200 km^2^ study area ranges from large agricultural fields to smaller tracts of farmland scattered with coniferous forests of various ages (Vasko et al. 2011; Kujala et al. 2024). Kestrels in our study area breed almost exclusively in nest‐boxes attached to barn walls (Korpimäki, 1983; Korpimäki and Wiehn 1998), reducing the likelihood of unrecorded nesting attempts.

We collected data of kestrel nesting attempts from 1985 until 2018. We inspected boxes for occupancy in mid‐May to late May and followed up nests in June to early July to confirm hatching and fledging. For each nesting attempt, we determined laying date by visiting each nest at multiple instances during the breeding season. If the clutch was incomplete during the first nest visit, laying date could be estimated precisely by assuming a two‐day egg‐laying interval (Piechocki 1982; Village 1990). For the vast majority of clutches, however, we estimated laying date by back‐dating from hatching dates, assuming that incubation lasts 30 days and kestrels start to incubate after laying their third egg (Village 1990; Korpimäki and Wiehn 1998). We recorded a total of 2454 nesting attempts in 1985–2018, of which we successfully determined laying date for 98%.

From 1985 to 2013, we caught breeding adult kestrels of both sexes at nests using swing‐door traps and either ringed or identified them from a pre‐existing ring during incubation or nestling period (see Vasko et al. 2011; Terraube et al. 2015 for more details on capture methods). We aged and sexed trapped kestrels based on plumage characteristics; at earliest, kestrels breed in their second year (hereafter SY), during which their post‐juvenile moult has progressed far enough to show sexual dimorphism. Two age classes are identifiable: juvenile tail feathers of SY individuals can be visually distinguished from moulted ones of after‐second‐year individuals (hereafter ASY; Forsman 1999). In the years of active parent trapping, the mean trapping efficiency (% of captured parents out of all nesting attempts) per year was 87% (SD = 8.3) for females and 79% (SD = 9.8) for males. In total, we observed 2846 kestrels (1402 females and 1037 males) breeding at the main study site during 1985–2013, and we encountered 476 of them (212 females and 264 males) across two or more years. At the initial encounter, most individuals (2239 or 79%) were aged as ASY; only 607 (21%) kestrels were encountered breeding as SY.

For estimating the density of the local vole population, we set snap traps for small mammals to 7–14 sample plots, located in western and central sites within the study area. At both sites, we established sample plots in four habitat types (cultivated field, fallow field, spruce‐dominated forest, pine‐dominated forest). In each sample plot, 50–100 Finnish mouse traps were set up for 3–4 nights and inspected once per day. Accumulating all trap sites for each trapping season, spanning from early to mid‐May, this resulted in 1200 to 2300 trap nights per season. We calculated vole indices as the total number of individuals from genera *Microtus* and *Myodes* per hundred trap nights (see Korpimäki et al. 2005 for more details on trapping procedure).

### 2.2 Winter Distribution of Finnish Kestrels

**FIGURE 1:**
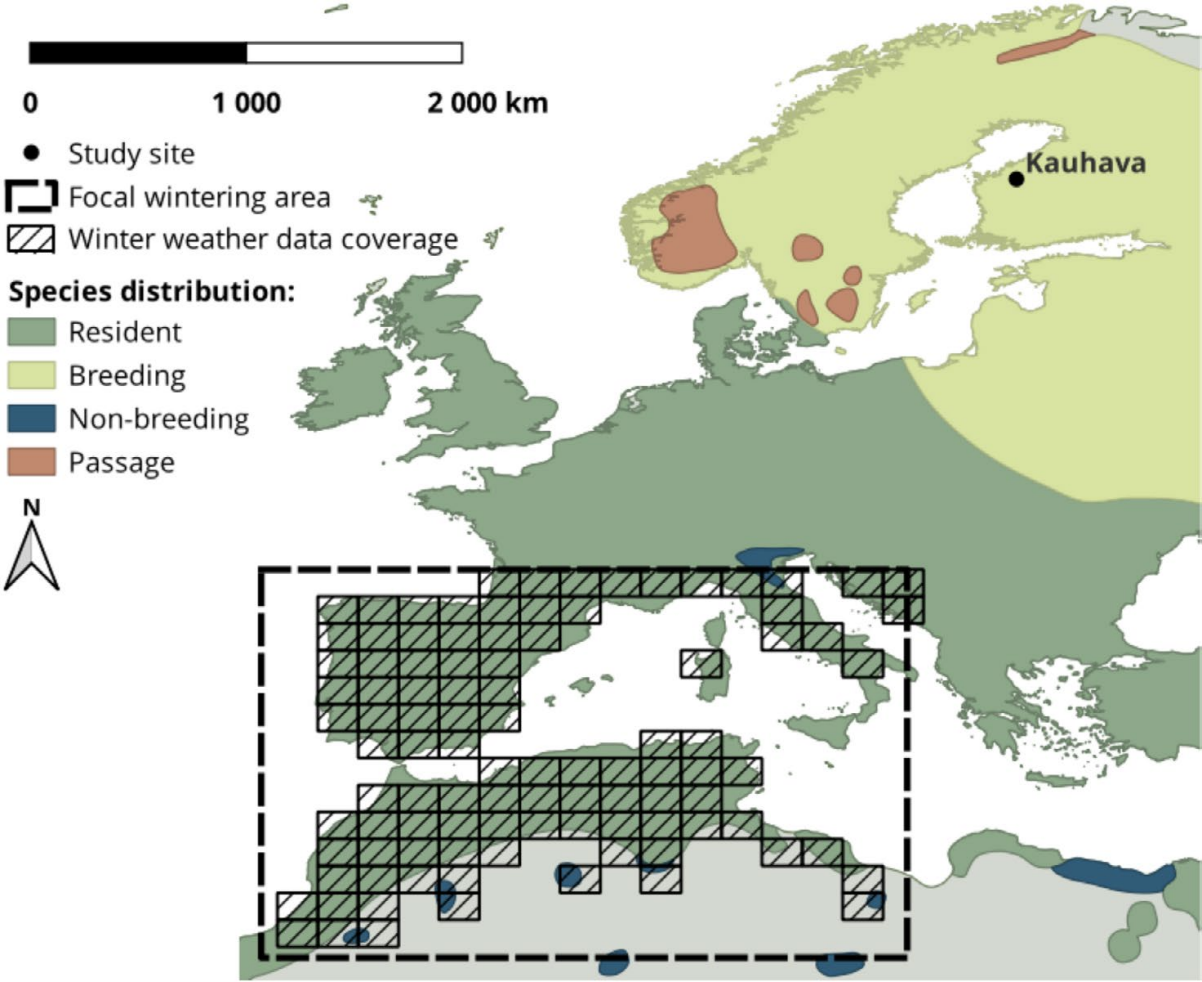
Map showing partial coverage of Europe and Mediterranean Africa and the distribution of Eurasian Kestrels within the displayed region (extracted from BirdLife International 2023), including the study area location (Kauhava, Finland; 63° N, 23° E) in which weather data were recorded during the breeding season, and the estimated focal wintering region (dashed rectangle) of our study population, spanning the area between 27° N–45° N and 12° W–18° E. Hashed grid shows the extent of HadEX3 data (Dunn et al. 2020) used to approximate weather conditions of winter months.

In order to assess environmental conditions on the kestrel's wintering grounds, we had to determine where kestrels breeding in Western Finland predominantly spent their winter. According to the ‘leapfrog migration’ hypothesis, North European kestrels migrate to Southern Europe and North Africa (Wallin et al. 1987). Based on ring recoveries gathered during 1913–2008 (779 recoveries outside of Finland in total; Saurola et al. 2013), kestrels ringed in Finland have been recovered mostly around the Mediterranean Sea (primarily in Spain, Italy, Morocco, Algeria and France) or in Central Europe (primarily in Germany, Switzerland, Austria and Hungary) during winter months. However, with ring recoveries alone it is impossible to confirm whether encountered kestrels had reached their ultimate wintering area or if they were recovered at a stopover site. We assume that Finnish kestrels overwinter more frequently in Southern Europe and North Africa than ring recoveries would infer, as recovery data are likely biassed towards areas with higher recovering and reporting probabilities (e.g., densely populated Central Europe) and therefore underestimating the proportion of kestrels wintering in Africa (e.g., Sahel zone). Acknowledging all aforementioned information, we have estimated the Mediterranean region to be our primary area of interest regarding the wintering grounds of our study population. Therefore, we have approximated the focal area for winter distribution to be between 27° N–45° N and 12° W–18° E (Figure 1).

### 2.3 Weather Variables

As weather indicators on the approximate main area for winter distribution, we extracted weather variables from the HadEX3 dataset (Dunn et al. 2020) hosted by Climdex (https://www.climdex.org/) corresponding to the focal wintering area between 27° N–45° N and 12° W–18° E. From this overall wintering area, we excluded uninhabitable regions, mainly desert, by limiting the extent of weather data to kestrel distribution range (extracted from BirdLife International 2023). From the remaining area, we computed averages for TNn and R10 for winter (December–February), representing the time when kestrels are primarily on their wintering grounds. We also considered the North Atlantic Oscillation (NAO) index of winter (Hurrell 1995) as an alternative indicator of large‐scale winter weather, but because it was strongly correlated with w‐R10 (*r* = −0.78) without having an effect on survival estimates in the preliminary models, we excluded it in favour of w‐R10. For summer weather data, we searched the HadEX3 dataset for TNn and R10 of summer months (June–August) recorded in Kauhava Airport, Finland (63° N, 23° E), obtaining corresponding weather indicators for the breeding season (when kestrel broods have hatched).

### 2.4 Statistical Methods

We performed two analyses to assess how environmental variables affect adult survival (in Cormack‐Jolly‐Seber models, CJS) and laying date (in linear mixed models, LMMs). We compared models using Akaike Information Criterion (AIC), with lower AIC indicating better model fit; out of the top models with a ΔAIC < 2, we report parameter estimates from the one with fewest parameters, considered as the most parsimonious one (Burnham and Anderson 2002).

First, to estimate survival (Phi, ‘Φ’) and resighting probability (rho, ‘ρ’) of breeding kestrels, we built CJS capture‐recapture models in R version 4.3.3 (R Core Team 2024) using the package *marked* (Laake et al. 2013). Goodness‐of‐fit tests were run with package *R2ucare* (Gimenez et al. 2018) and tests for overdispersion in programme MARK (White and Burnham 1999). We ran 104 candidate models (Table S1) with different combinations of seven explanatory variables and two of their interactions to predict survival. Encounter occasions were annual, and all captures occurred between April 14th and August 1st. When predicting survival from breeding season *t* to *t + 1*, we considered the following time‐varying explanatory variables: vole index, s‐TNn and s‐R10 of year *t*, and w‐TNn and w‐R10 of winter following *t* (December of year *t* and January–February of *t + 1*). We fitted w‐R10 as a quadratic variable to represent both extreme drought and rainfall occurring in Mediterranean winter; s‐R10 was fitted as linear, as water availability is greater in boreal biomes than in the Mediterranean (Ruiz‐Benito et al. 2014). Lastly, we included age (factor with two levels: SY and ASY) as a covariate. We also considered interactions between age and two rainfall‐related variables (s‐R10 and w‐R10^2^) to examine the differences in responses between age classes. All numeric variables were scaled and mean‐centred. We tested all covariates for pairwise correlations: no highly correlated (|*r*| > 0.4) predictor pairs emerged.

As expected from a species with generally high population turnover rates (Village 1990), significant results emerged in the test for transience (i.e., permanent emigration, test3.sr, *p* < 0.01). To account for the frequency of permanent emigration, we implemented a categorical variable (time since marking, TSM) representing encounter occasion (0 = first encounter, 1 = all subsequent capture occasions), allowing the model to assign higher estimates of apparent survival to those individuals who have been encountered at least twice and can be thus assumed as non‐transient (Pradel et al. 1997). This variable was included in all models of *Φ*. No other deviations from model assumptions emerged in goodness‐of‐fit tests. Because site fidelity in males is notably greater than in females (Vasko et al. 2011; Terraube et al. 2015; Holte et al. 2016), we fitted resighting probability as sex‐specific (factor). The dispersion parameter *ĉ* indicated no overdispersion (median *ĉ* = 1.30).

To predict laying date, we computed and ranked linear mixed models using packages *nlme* (Pinheiro et al. 2023) and *MuMIn* (Bartón 2023). To examine the carry‐over effects of wintering conditions on laying date (continuous, 3–78; 1 = 1st of April), we considered TNn and R10 of preceding winter as explanatory variables. We considered fitting w‐R10 as a quadratic term, but because its relationship with laying date appeared linear during initial data exploration, we included w‐R10 only as a linear predictor in the final set of candidate models. Additionally, we assigned vole index to each candidate model as a predictor to account for variation in breeding phenology associated with food availability, with kestrels typically initiating egg‐laying earlier in years with high vole abundance (Korpimäki and Wiehn 1998). We also included the interactions between vole index and w‐TNn and w‐R10 to test whether the potential carry‐over effect of winter weather was relative to food availability following migration, expecting that prey abundance could enhance migration recovery and therefore compensate for carry‐over effects. Finally, we included year as a random effect to account for unexplained interannual variation and the non‐independence of breeding attempts occurring during the same year.

We assessed model fit by visually examining residual distribution. In preliminary diagnostics plots, residuals showed decreasing variance towards greater fitted values, indicating heteroskedasticity. To accommodate this, we included a power variance function structure with the variance covariate equal to the fitted values, allowing residual variance to scale as an estimated power of the mean. The power parameter (δ = −1.04) was estimated during model fitting. We tested for spatial autocorrelation in laying date with Moran's I test using package *ape* (Paradis and Schliep 2019). As Moran's I test indicated significant autocorrelation (*p* < 0.001), we included an exponential spatial correlation structure in the laying date model. Because multiple observations occurred at the same nest‐box locations, we added a small jitter of 10^−6^ degrees to duplicate coordinates using package *geoR* (Ribeiro Jr. et al. 2024), ensuring all distances in the spatial distance matrix were non‐zero.

## 3 Results

### 3.1 Survival Rate in Relation to Environmental Conditions

**FIGURE 2:**
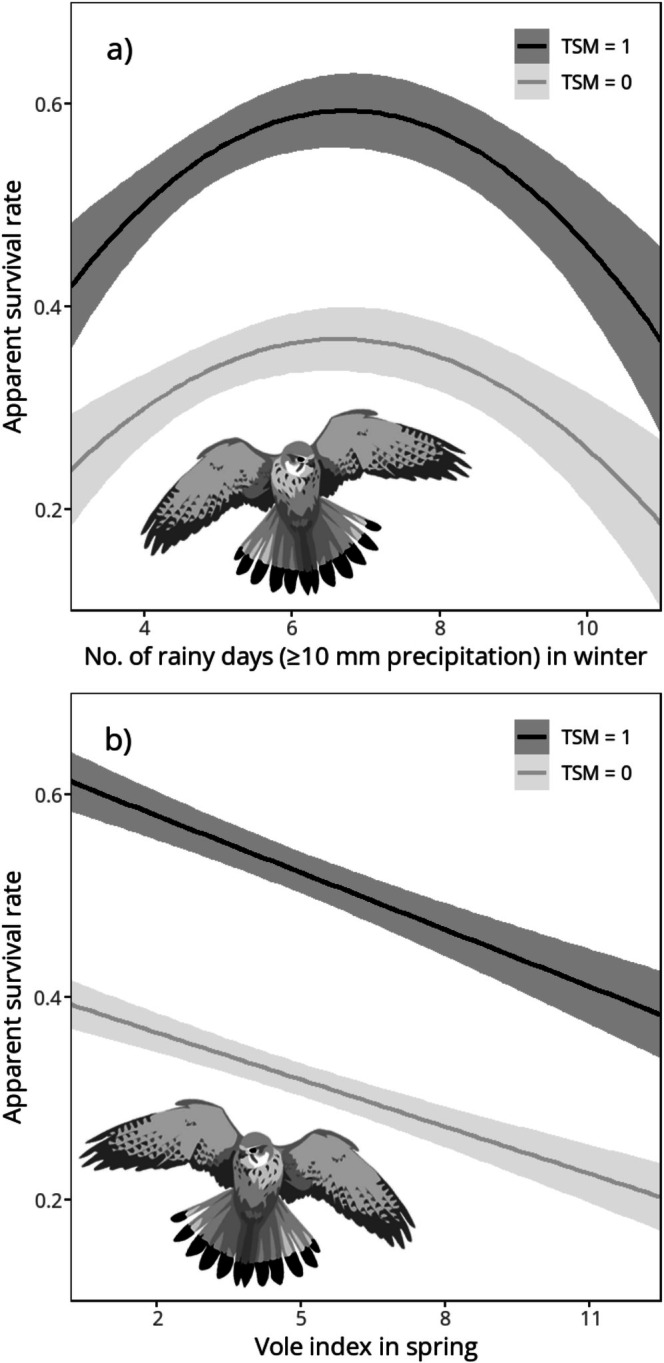
Cormack‐Jolly‐Seber (CJS) effect sizes and 95% confidence intervals from the most parsimonious model of annual apparent survival rate of kestrels encountered in 1985–2013 in relation to (a) wintertime frequency of rainy days (precipitation ≥ 10 mm) and (b) vole abundance (no. of voles per hundred trap nights) of preceding spring. Grey line displays effect sizes for survival estimates following the first encounter of an individual (TSM = 0), black line displays effect sizes for survival estimates for subsequent capture intervals (TSM = 1).

Estimated survival rate (i.e., *Φ* estimated as constant) of breeding kestrels was 0.45 (95% CI: 0.42, 0.48) during 1985–2013. When assigning separate estimates of Φ for first and subsequent encounters, the estimate was 0.31 (95% CI: 0.27, 0.35) following first encounter and 0.52 (95% CI: 0.48, 0.55) for subsequent encounter intervals. No linear trend for the time span of 1985–2013 was detected by predicting survival with year as a continuous explanatory variable (95% CI: −0.01, 0.01). Seven models with ∆AIC < 2 emerged (Table 1), of which the most parsimonious based on AIC and the number of parameters (*K*) included vole index of preceding spring and w‐R10 (linear and quadratic) as predictors of survival (Table 2). The relationship between survival and w‐R10 was primarily quadratic, with both extremely high and low numbers of rainy days being associated with reduced survival (Figure 2a). Vole abundance appeared to correlate negatively with survival (Figure 2b). Summer TNn and age were featured in the first‐ranked (smallest AIC) model, but the confidence intervals of their effect sizes included zero (95% CI for s‐TN: −0.02, 0.24; 95% CI for age (ASY as reference level): −0.61, 0.06), and model AIC was only slightly smaller (∆AIC = 1.34) compared to the most parsimonious model. Resighting probability differed between sexes: males were more likely to be re‐encountered (estimated *ρ* = 0.52; 95% CI: 0.46, 0.58) than females (estimated *ρ* = 0.23; 95% CI: 0.19, 0.28; estimates from the most parsimonious model).

**TABLE 1:** Top (∆AIC < 2) seven CJS models predicting survival (*Φ*) in kestrels, based on the capture histories of 2728 individuals breeding in Finland.

Model		*K*	AIC	∆AIC	*ω*_i_
1.	Φ (TSM + age + s‐TNn + w‐R10^2^ + w‐R10 + voles) *ρ* (sex)	9	3742.96	0.00	0.12
2.	Φ (TSM + s‐TNn + w‐R10^2^ + w‐R10 + voles) *ρ* (sex)	8	3743.56	0.60	0.09
3.	Φ (TSM + age + w‐R10^2^ + w‐R10 + voles) *ρ* (sex)	8	3743.72	0.76	0.08
**4**.	**Φ (TSM + w‐R10** ^**2**^ **+ w‐R10 + voles) ρ (sex)**	**7**	**3744.29**	**1.34**	**0.06**
5.	Φ (TSM + age + s‐TNn + w‐TNn + w‐R10^2^ + w‐R10 + voles) *ρ* (sex)	10	3744.77	1.82	0.05
6.	Φ (TSM + age + s‐TNn + w‐R10^2^ + w‐R10 + voles + age × w‐R10^2^) *ρ* (sex)	10	3744.89	1.93	0.05
7.	Φ (TSM + age + s‐TNn + w‐R10^2^ + s‐R10 + w‐R10 + voles) *ρ* (sex)	10	3744.95	1.99	0.05

*Note:* *K* refers to the number of parameters in each model, *ω*_i_ to model weight. age, age class (second‐year, as opposed to after second‐year); s‐R10, number of rainy days in summer; s‐TNn, minimum temperature of summer; TSM, time since marking (0 = first encounter, 1 = all subsequent capture occasions); voles, vole index in preceding May; w‐R10, number of rainy days (with ≥ 10 mm precipitation) in winter; w‐R10^2^, squared w‐R10; w‐TNn, minimum temperature of winter. For predicting resighting probability (*ρ*), each model has identical variable composition, comprising sex. The most parsimonious model based on *K* within ∆AIC < 2 range is shown in bold.

**TABLE 2:** Model estimates of the most parsimonious CJS model based on AIC and *K* predicting survival (*Φ*) and resighting probability (*ρ*) in kestrels, based on the capture histories of 2728 individuals breeding in Finland.

Effect	Estimate	SE	95% CI
Φ: TSM (1)	0.89	0.13	(0.63, 1.14)
Φ: voles	−0.29	0.06	(−0.41, −0.17)
Φ: w‐R10	0.02	0.06	(−0.11, 0.14)
Φ: w‐R10^2^	−0.16	0.04	(−0.24, −0.07)
Φ: Intercept	−0.60	0.11	(−0.81, −0.39)
ρ: sex (male)	1.29	0.14	(1.00, 1.57)
ρ: Intercept	−1.20	0.13	(−1.45, −0.95)

*Note:* Effect sizes are presented alongside their standard errors and 95% confidence intervals. See Table 1 for abbreviation definitions.

### 3.2 Winter Precipitation and Timing of Egg‐Laying

**FIGURE 3:**
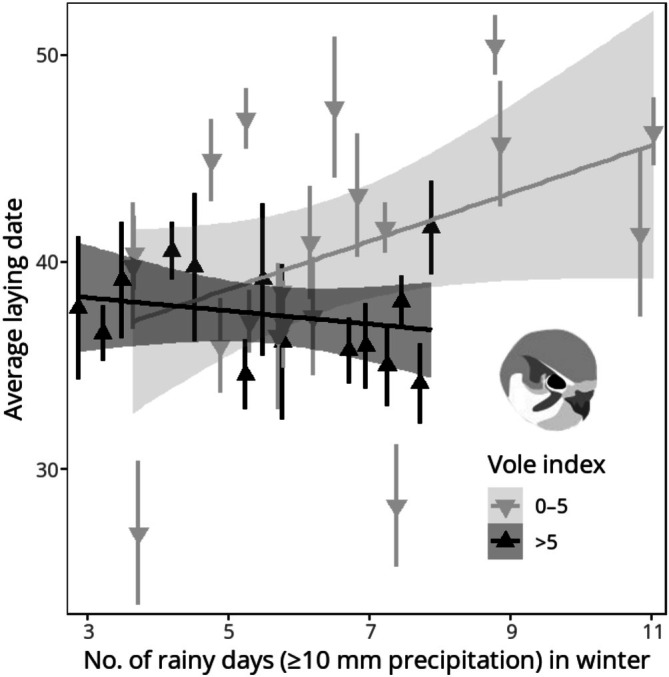
The relationship between the yearly average laying date (count from the beginning of April, 1 = 1st of April) of kestrels and the frequency of rainy days (precipitation ≥ 10 mm) in the preceding winter during years of low (0–5, *n* = 20) and high (> 5, *n* = 14) vole abundance (no. of voles per hundred trap nights) in spring during 1985–2018. Annual averages and regressions are presented with 95% confidence intervals.

The annual average of laying date (mean = 9th of May, SD = 5.01 d) did not change (*r*_S_ = 0.07, *p* = 0.71) during 1985–2018. Out of nine candidate models, two emerged within ΔAIC < 2 range, with the top‐ranked model being the most parsimonious (Table S2). The best model predicting laying date included vole index, w‐R10, and their interaction as explanatory variables (Table 3). The effect of w‐R10 on laying date was conditional on vole index: the interaction term was negative, meaning that a high frequency of rainy days in winter delayed laying during years of low vole abundance (Figure 3).

**TABLE 3:** Linear mixed model parameter estimates of variables predicting laying date (*n* = 2411) in kestrels, presented with their standard errors (SE) and 95% confidence intervals (CI).

Effect	Estimate	SE	95% CI
w‐R10	0.77	0.72	(−0.69, 2.24)
voles	−2.09	0.68	(−3.48, −0.69)
w‐R10 × voles	−2.22	0.88	(−4.02, −0.42)
Intercept	39.03	0.68	(37.70, 40.36)

*Note:* See Table 1 for other abbreviation definitions.

## 4 Discussion

### 4.1 Extreme Weather Associates With Decreased Survival

To the best of our knowledge, our study provides the first evidence of impacts of climate extremes on adult survival rate in a long‐distance migratory species breeding in the Nordics, perhaps because possible impacts of climate change‐induced extremes in weather conditions on biological systems have only recently received attention among ecologists (e.g., Bailey and van de Pol 2016; Murali et al. 2023; Preston‐Allen et al. 2024). Specifically, as per our prediction, both extremely low and high numbers of rainy days during winter were associated with reduced survival in kestrels. Reduced survival in kestrels during drought is likely caused by the scarcity of prey animals arising from limited vegetation growth (Gouveia et al. 2016; Chidodo et al. 2020; Klein et al. 2020). Concurrently, kestrels are unable to forage efficiently during periods of rainfall and are likely relying on sparse hunting opportunities to sustain themselves during seasons of frequent or continuous rainfall (Rijnsdorp et al. 1981; Village 1990). Previously, drought‐originated reductions in survival have been shown to occur in red kites ( *Milvus milvus* ) in Mediterranean marshland biome (Sergio et al. 2022). In addition, extreme precipitation or drought in African wintering areas have been reported to similarly impact the survival of two highly mobile aerial insectivores, the pallid swift ( *Apus pallidus* ) and common swift ( *Apus apus* ), breeding in Italy (Boano et al. 2020).

Contrary to our predictions, seasonal minimum temperature of Mediterranean winter had no apparent effect on kestrel survival. As our weather variables were rough estimates extracted from a sizeable area without precise knowledge of where the primary wintering grounds of our study population are located, the variables used as predictors do not necessarily reflect the overall weather conditions faced by long‐distance migratory kestrels. Furthermore, the absolute minimum of seasonal temperature does not indicate the length of cold spells within the season. A more robust indicator of large‐scale temperature patterns could demonstrate a relationship between winter temperatures and survival; such a relationship has been documented in American kestrels ( *Falco sparverius* ), with warmer winters improving their survival rates (Callery et al. 2022).

The influence of weather in the breeding areas on the foraging ability, breeding performance and body condition has been studied extensively in kestrels and other diurnal raptors (e.g., Rodríguez and Bustamante 2003; Anctil et al. 2014; Kujala et al. 2024). In particular, heavy precipitation appears to prevent efficient foraging, resulting in reduced brood size. However, none of the examined variables representing the weather in the breeding season influenced the survival of breeding kestrels. This disparity could result from parent kestrels adjusting their breeding efforts according to prevailing environmental conditions precisely enough to avoid compromising their survival likelihood when facing adverse weather (Korpimäki and Rita 1996; Dijkstra et al. 1990).

For migrant bird species, mortality generally appears to peak during migration seasons (Newton 2007; Rockwell et al. 2017). Additionally, elevated mortality rates have been reported to occur in late summer in some migratory raptors (Klaassen et al. 2014). According to our results, the influence of adverse weather experienced during the peak reproductive season appears insignificant regarding annual survival in kestrels. Contrarily, severe weather in non‐breeding areas can affect individuals' ability to maintain a healthy condition during winter and successfully prepare for spring migration and the upcoming breeding season. While autumn migration appears as a bottleneck to juveniles migrating for the first time (Buechley et al. 2021), spring migration has been reported as a particularly risky migration period for adults of several raptor species in the Palearctic‐African migratory system (Klaassen et al. 2014).

### 4.2 Survival in Relation to Food Conditions and Age

Contrary to our predictions, there was a strong negative association between springtime vole abundances and survival of kestrels. Opposite responses in survival have been recorded in other vole specialists breeding in the boreal zone. In male Tengmalm's owls ( *Aegolius funereus* ), survival probability is positively correlated with vole density (Hakkarainen et al. 2002). Overwinter survival of Eurasian pygmy owls ( *Glaucidium passerinum* ) is positively related to prey biomass in food‐stores in the preceding autumn (Korpimäki et al. 2025), with food‐store sizes being positively associated with vole abundance (Masoero et al. 2018). In tawny owls ( *Strix aluco* ), vole abundance had no obvious effect on survival (Karell et al. 2009) while for Ural owls ( *Strix uralensis* ) it was positively associated with survival probability of first‐year individuals (Saurola and Francis 2018). Unlike Finnish kestrels, these owl species stay over winter in the boreal zone, and their annual survival is thus strongly affected by overwinter availability of voles (Hakkarainen et al. 2002).

Regarding kestrels, the negative association between vole abundance and survival is most likely due to increased numbers of transient individuals (i.e., individuals which emigrate permanently after first encounter) breeding during years of high vole abundance, rather than high vole abundance reducing true survival. As dense vole populations attract more breeding kestrels to an area, many of those individuals should either disperse or fail to reattempt breeding after vole abundances diminish (Korpimäki and Norrdahl 1991; Vasko et al. 2011), resulting in seemingly low survival rates following peak vole densities. On the other hand, springtime food availability is strongly associated with reproductive effort and success of kestrels (Tolonen and Korpimäki 1994; Korpimäki and Wiehn 1998). Accordingly, the non‐breeding season following a plentiful vole year should feature a high post‐breeding number of individuals. As survival during non‐breeding periods appears density dependent in several migratory species (reviewed by Newton 2008), high population densities of kestrels following vole population peaks could also lead to a temporary increase in mortality, when limited resources are distributed between an increased number of conspecifics (McCabe et al. 2022) and heterospecifics subsisting on similar food resources (Korpimäki 1987). Furthermore, producing large broods may be particularly costly for breeding males, who are the primary providers, potentially resulting in a sex‐dependent effect of reproductive cost on survival (Bühler et al. 2024).

We found no disparity in apparent survival rate between the two age groups (SY and ASY) and no interactions between age and precipitation effects. As individuals can be aged as SY only on their first breeding attempt, we believe that any potential reduction in their true survival rate was conflated with the TSM variable accounting for transience. While the positive influence of winter precipitation appears to be age‐specific in the lesser kestrel ( *Falco naumanni* ), affecting only first‐year individuals (Mihoub et al. 2010), there was no such division in our kestrel population. Being smaller and more specialised predators, lesser kestrels might require greater skill and experience to utilise alternative food sources compared to kestrels. These contrasting results could also reflect other niche dissimilarities, such as differing microhabitat preferences or natal dispersal distances.

Considering the influence of weather in wintering grounds on survival, there are potential climate‐related threats concerning migratory kestrel populations. Drought spells have become more frequent in the Mediterranean area and North Africa (Cook et al. 2018), while predicted temperature and precipitation trends are anticipated to accelerate desertification across these wintering areas of kestrels (Carvalho et al. 2022). Simultaneously, extreme precipitation events occur increasingly in this region (Benabdelouahab et al. 2020). With these trends, the availability of suitable winter territories and even large‐scale migratory patterns of European kestrels could be altered in the future.

### 4.3 Frequent Winter Precipitation Indicates Delayed Laying During Poor Vole Years

We found that frequent precipitation in the Mediterranean wintering grounds delayed the initiation of egg‐laying in North European kestrels, but this effect was conditional on the abundance of primary prey (voles) in the breeding area. The interaction term between w‐R10 and vole indices suggests that during years with abundant springtime vole populations, the carry‐over effect of disadvantageous weather conditions is negated. This implies that when food availability at the breeding grounds reaches a certain threshold, arriving kestrels are inclined to initiate breeding early instead of strengthening their body condition beforehand. Alternatively, when met with prey abundance after migration, even individuals in poor condition might regain their energy reserves without additional delay, as preceding food insecurity enhances energetic efficiency and mass gain (Jönsson et al. 1999; Bateson et al. 2021). While the delays in laying date could simply be indicative of late arrival to the breeding grounds, the carry‐over effect of postponing the initiation of breeding when faced with unfavourable environmental conditions preceding the breeding season has been documented in resident and migratory bird species alike (Gill et al. 2001; Saino et al. 2004; López‐Peinado and López‐López 2024).

## 5 Conclusions

Our results, based on a robust, long‐term dataset, demonstrate the causality between Mediterranean climate and population‐level responses in migrant kestrels breeding in North Europe, representing a rarely studied long‐distance migratory system. We conclude that weather extremes in wintering areas are more acutely affecting annual adult survival in migratory kestrels than weather conditions in breeding grounds, thus providing the first evidence of impacts of climate extremes on survival rate of North European long‐distance migrants. Moreover, the effects of adverse weather are carried over to the breeding season. In poor food conditions, this carryover effect manifests as a delay in the initiation of egg‐laying. As global climate warming will likely increase the frequency of weather anomalies across the wintering grounds while simultaneously repressing the abundances of main foods of kestrels, the population dynamics of migratory kestrels are prone to change. Being partial migrants, kestrels could potentially respond to some of the alterations in their environment by changing their migratory strategies and wintering range. However, as many of their global populations dwindle (Constantini and Dell'Omo 2020; Shaw et al. 2024), maintaining the stability of the larger populations remains relevant. Further conservation measures will be necessary to preserve both quality and quantity of their wintering habitats.

## Author Contributions

**Inga Kujala:** conceptualization (supporting), formal analysis (lead), methodology (equal), writing – original draft (lead), writing – review and editing (supporting). **Carina Nebel:** formal analysis (supporting), methodology (equal), writing – review and editing (equal). **Hannu Pöysä:** writing – review and editing (equal). **Erkki Korpimäki:** conceptualization (lead), investigation (lead), methodology (equal), writing – review and editing (equal).

## Conflicts of Interest

The authors declare no conflicts of interest.

## Supporting information


**Appendix S1:** ece372741‐sup‐0001‐supinfo.docx.

## Data Availability

The data that support the findings of this study are openly available in Dryad at http://doi.org/10.5061/dryad.gqnk98t2m.
