# Supplementary material for: Weather Extremes in the Mediterranean Winter Are Associated With Reduced Apparent Survival and Delayed Initiation of Egg‐Laying in a Migratory Raptor
Source: Ecol Evol. 2025 Dec 18;15(12):e72741. doi: 10.1002/ece3.72741 (PMC12714412; doi:10.1002/ece3.72741)
Supplement: Supplementary file 1 — Appendix S1: ece372741‐sup‐0001‐supinfo.docx. [file ECE3-15-e72741-s001.docx]

**Supplementary Information**

**Table S1.** CJS models ranked by AIC predicting apparent survival (Φ) in kestrels, based on the capture histories of 2,728 individuals breeding in Finland. *K* refers to the number of parameters in each model, ω_i_ to model weight. TSM = time since marking (0 = first encounter, 1 = all subsequent capture occasions), w-TNn = minimum temperature of winter, s-TNn = minimum temperature of summer, w-R10 = number of rainy days (with ≥ 10 mm precipitation) in winter, w-R10^2^ = squared w-R10, s-R10 = number of rainy days in summer. For predicting resighting probability (ρ), each model has identical variable composition, comprising sex.

| Model | | *K* | AIC | ∆AIC | ω_i_ |
| --- | --- | --- | --- | --- | --- |
| 1. | Φ (TSM + age + s-TNn + w-R10^2^ + w-R10 + voles) ρ (sex) | 9 | 3742.96 | 0.00 | 0.12 |
| 2. | Φ (TSM + s-TNn + w-R10^2^ + w-R10 + voles) ρ (sex) | 8 | 3743.56 | 0.60 | 0.09 |
| 3. | Φ (TSM + age + w-R10^2^ + w-R10 + voles) ρ (sex) | 8 | 3743.72 | 0.76 | 0.08 |
| 4. | Φ (TSM + w-R10^2^ + w-R10 + voles) ρ (sex) | 7 | 3744.29 | 1.34 | 0.06 |
| 5. | Φ (TSM + age + s-TNn + w-TNn + w-R10^2^ + w-R10 + voles) ρ (sex) | 10 | 3744.77 | 1.82 | 0.05 |
| 6. | Φ (TSM + age + s-TNn + w-R10^2^ + w-R10 + voles + age * w-R10^2^) ρ (sex) | 10 | 3744.89 | 1.93 | 0.05 |
| 7. | Φ (TSM + age + s-TNn + w-R10^2^ + s-R10 + w-R10 + voles) ρ (sex) | 10 | 3744.95 | 1.99 | 0.05 |
| 8. | Φ (TSM + age + w-TNn + w-R10^2^ + w-R10 + voles) ρ (sex) | 9 | 3745.04 | 2.08 | 0.04 |
| 9. | Φ (TSM + s-TNn + w-TNn + w-R10^2^ + w-R10 + voles) ρ (sex) | 9 | 3745.38 | 2.43 | 0.04 |
| 10. | Φ (TSM + s-TNn + w-R10^2^ + s-R10 + w-R10 + voles) ρ (sex) | 9 | 3745.55 | 2.60 | 0.03 |
| 11. | Φ (TSM + w-TNn + w-R10^2^ + w-R10 + voles) ρ (sex) | 8 | 3745.63 | 2.67 | 0.03 |
| 12. | Φ (TSM + age + w-R10^2^ + w-R10 + voles + age * w-R10^2^) ρ (sex) | 9 | 3745.68 | 2.73 | 0.03 |
| 13. | Φ (TSM + age + w-R10^2^ + s-R10 + w-R10 + voles) ρ (sex) | 9 | 3745.71 | 2.75 | 0.03 |
| 14. | Φ (TSM + w-R10^2^ + s-R10 + w-R10 + voles) ρ (sex) | 8 | 3746.28 | 3.33 | 0.02 |
| 15. | Φ (TSM + age + s-TNn + w-R10^2^ + s-R10 + w-R10 + voles + age * s-R10) ρ (sex) | 11 | 3746.28 | 3.33 | 0.02 |
| 16. | Φ (TSM + age + s-TNn + w-TNn + w-R10^2^ + w-R10 + voles + age * w-R10^2^) ρ (sex) | 11 | 3746.71 | 3.75 | 0.02 |
| 17. | Φ (TSM + age + s-TNn + w-TNn + w-R10^2^ + s-R10 + w-R10 + voles) ρ (sex) | 11 | 3746.71 | 3.76 | 0.02 |
| 18. | Φ (TSM + age + s-TNn + w-R10^2^ + s-R10 + w-R10 + voles + age * w-R10^2^) ρ (sex) | 11 | 3746.88 | 3.92 | 0.02 |
| 19. | Φ (TSM + age + w-R10^2^ + s-R10 + w-R10 + voles + age * s-R10) ρ (sex) | 10 | 3746.98 | 4.03 | 0.02 |
| 20. | Φ (TSM + age + w-TNn + w-R10^2^ + s-R10 + w-R10 + voles) ρ (sex) | 10 | 3746.99 | 4.04 | 0.02 |
| 21. | Φ (TSM + age + w-TNn + w-R10^2^ + w-R10 + voles + age * w-R10^2^) ρ (sex) | 10 | 3747.01 | 4.05 | 0.02 |
| 22. | Φ (TSM + s-TNn + w-TNn + w-R10^2^ + s-R10 + w-R10 + voles) ρ (sex) | 10 | 3747.33 | 4.37 | 0.01 |
| 23. | Φ (TSM + w-TNn + w-R10^2^ + s-R10 + w-R10 + voles) ρ (sex) | 9 | 3747.59 | 4.63 | 0.01 |
| 24. | Φ (TSM + age + w-R10^2^ + s-R10 + w-R10 + voles + age * w-R10^2^) ρ (sex) | 10 | 3747.67 | 4.72 | 0.01 |
| 25. | Φ (TSM + age + s-TNn + voles) ρ (sex) | 7 | 3747.92 | 4.96 | 0.01 |
| 26. | Φ (TSM + age + s-TNn + w-R102 + s-R10 + w-R10 + voles + age * w-R10^2^ + age * s-R10) ρ (sex) | 12 | 3747.95 | 5.00 | 0.01 |
| 27. | Φ (TSM + age + s-TNn + w-TNn + w-R10^2^ + s-R10 + w-R10 + voles + age * s-R10) ρ (sex) | 12 | 3748.07 | 5.12 | 0.01 |
| 28. | Φ (TSM + age + w-TNn + w-R10^2^ + s-R10 + w-R10 + voles + age * s-R10) ρ (sex) | 11 | 3748.33 | 5.37 | 0.01 |
| 29. | Φ (TSM + s-TNn + voles) ρ (sex) | 6 | 3748.42 | 5.46 | 0.01 |
| 30. | Φ (TSM + age + s-TNn + w-TNn + w-R10^2^ + s-R10 + w-R10 + voles + age * w-R10^2^) ρ (sex) | 12 | 3748.66 | 5.70 | 0.01 |
| 31. | Φ (TSM + age + w-R10^2^ + s-R10 + w-R10 + voles + age * w-R10^2^ + age * s-R10) ρ (sex) | 11 | 3748.71 | 5.76 | 0.01 |
| 32. | Φ (TSM + age + w-TNn + w-R10^2^ + s-R10 + w-R10 + voles + age * w-R10^2^) ρ (sex) | 11 | 3748.97 | 6.01 | 0.01 |
| 33. | Φ (TSM + age + s-TNn + s-R10 + voles) ρ (sex) | 8 | 3749.35 | 6.40 | 0.01 |
| 34. | Φ (TSM + age + s-TNn + w-TNn + voles) ρ (sex) | 8 | 3749.70 | 6.74 | 4E-03 |
| 35. | Φ (TSM + age + s-TNn + w-TNn + w-R10^2^ + s-R10 + w-R10 + voles + age * w-R10^2^ + age * s-R10) ρ (sex) | 13 | 3749.77 | 6.81 | 4E-03 |
| 36. | Φ (TSM + s-TNn + s-R10 + voles) ρ (sex) | 7 | 3749.85 | 6.90 | 4E-03 |
| 37. | Φ (TSM + age + w-TNn + w-R10^2^ + s-R10 + w-R10 + voles + age * w-R10^2^ + age * s-R10) ρ (sex) | 12 | 3750.09 | 7.14 | 3E-03 |
| 38. | Φ (TSM + s-TNn + w-TNn + voles) ρ (sex) | 7 | 3750.19 | 7.24 | 3E-03 |
| 39. | Φ (TSM + age + s-TNn + s-R10 + voles + age * s-R10) ρ (sex) | 9 | 3750.80 | 7.84 | 2E-03 |
| 40. | Φ (TSM + age + s-TNn + w-TNn + s-R10 + voles) ρ (sex) | 9 | 3750.93 | 7.98 | 2E-03 |
| 41. | Φ (TSM + s-TNn + w-TNn + s-R10 + voles) ρ (sex) | 8 | 3751.43 | 8.47 | 2E-03 |
| 42. | Φ (TSM + age + s-TNn + w-TNn + s-R10 + voles + age * s-R10) ρ (sex) | 10 | 3752.34 | 9.39 | 1E-03 |
| 43. | Φ (TSM + age + s-R10 + voles) ρ (sex) | 7 | 3757.35 | 14.39 | 9E-05 |
| 44. | Φ (TSM + age + voles) ρ (sex) | 6 | 3757.62 | 14.66 | 8E-05 |
| 45. | Φ (TSM + s-R10 + voles) ρ (sex) | 6 | 3757.71 | 14.75 | 8E-05 |
| 46. | Φ (TSM + voles) ρ (sex) | 5 | 3757.95 | 15.00 | 7E-05 |
| 47. | Φ (TSM + age + s-R10 + voles + age * s-R10) ρ (sex) | 8 | 3758.83 | 15.87 | 4E-05 |
| 48. | Φ (TSM + age + w-TNn + s-R10 + voles) ρ (sex) | 8 | 3759.08 | 16.12 | 4E-05 |
| 49. | Φ (TSM + w-TNn + s-R10 + voles) ρ (sex) | 7 | 3759.43 | 16.48 | 3E-05 |
| 50. | Φ (TSM + age + w-TNn + voles) ρ (sex) | 7 | 3759.60 | 16.64 | 3E-05 |
| 51. | Φ (TSM + w-TNn + voles) ρ (sex) | 6 | 3759.93 | 16.98 | 3E-05 |
| 52. | Φ (TSM + age + w-TNn + s-R10 + voles + age * s-R10) ρ (sex) | 9 | 3760.52 | 17.57 | 2E-05 |
| 53. | Φ (TSM + w-R10^2^ + w-R10) ρ (sex) | 6 | 3763.72 | 20.77 | 4E-06 |
| 54. | Φ (TSM + age + w-R10^2^ + w-R10) ρ (sex) | 7 | 3763.72 | 20.77 | 4E-06 |
| 55. | Φ (TSM + age + w-TNn + w-R10^2^ + w-R10) ρ (sex) | 8 | 3764.41 | 21.46 | 3E-06 |
| 56. | Φ (TSM + w-TNn + w-R10^2^ + w-R10) ρ (sex) | 7 | 3764.45 | 21.49 | 3E-06 |
| 57. | Φ (TSM + age + w-R10^2^ + s-R10 + w-R10) ρ (sex) | 8 | 3764.80 | 21.84 | 2E-06 |
| 58. | Φ (TSM + w-R102 + s-R10 + w-R10) ρ (sex) | 7 | 3764.82 | 21.87 | 2E-06 |
| 59. | Φ (TSM + age + w-R10^2^ + s-R10 + w-R10 + age * s-R10) ρ (sex) | 9 | 3765.56 | 22.60 | 2E-06 |
| 60. | Φ (TSM + s-TNn + w-R10^2^ + w-R10) ρ (sex) | 7 | 3765.66 | 22.71 | 1E-06 |
| 61. | Φ (TSM + age + s-TNn + w-R10^2^ + w-R10) ρ (sex) | 8 | 3765.67 | 22.71 | 1E-06 |
| 62. | Φ (TSM + age + w-R10^2^ + w-R10 + age * w-R10^2^) ρ (sex) | 8 | 3765.71 | 22.76 | 1E-06 |
| 63. | Φ (TSM + age + s-TNn + w-TNn + w-R10^2^ + w-R10) ρ (sex) | 9 | 3765.98 | 23.02 | 1E-06 |
| 64. | Φ (TSM + s-TNn + w-TNn + w-R10^2^ + w-R10) ρ (sex) | 8 | 3766.01 | 23.05 | 1E-06 |
| 65. | Φ (TSM + age + w-TNn + w-R10^2^ + s-R10 + w-R10) ρ (sex) | 9 | 3766.10 | 23.15 | 1E-06 |
| 66. | Φ (TSM + w-TNn + w-R10^2^ + s-R10 + w-R10) ρ (sex) | 8 | 3766.15 | 23.19 | 1E-06 |
| 67. | Φ (TSM + age + w-TNn + w-R10^2^ + w-R10 + age * w-R10^2^) ρ (sex) | 9 | 3766.40 | 23.44 | 1E-06 |
| 68. | Φ (TSM + age + s-TNn + w-R10^2^ + s-R10 + w-R10) ρ (sex) | 9 | 3766.64 | 23.68 | 9E-07 |
| 69. | Φ (TSM + s-TNn + w-R10^2^ + s-R10 + w-R10) ρ (sex) | 8 | 3766.66 | 23.70 | 9E-07 |
| 70. | Φ (TSM + age + w-R10^2^ + s-R10 + w-R10 + age * w-R10^2^) ρ (sex) | 9 | 3766.79 | 23.83 | 8E-07 |
| 71. | Φ (TSM + age + w-TNn + w-R10^2^ + s-R10 + w-R10 + age * s-R10) ρ (sex) | 10 | 3766.86 | 23.90 | 8E-07 |
| 72. | Φ (TSM + age + w-R10^2^ + s-R10 + w-R10 + age * w-R10^2^ + age * s-R10) ρ (sex) | 10 | 3767.29 | 24.34 | 6E-07 |
| 73. | Φ (TSM + age + s-TNn + w-R10^2^ + s-R10 + w-R10 + age * s-R10) ρ (sex) | 10 | 3767.39 | 24.44 | 6E-07 |
| 74. | Φ (TSM + age + s-TNn + w-TNn + w-R10^2^ + s-R10 + w-R10) ρ (sex) | 10 | 3767.64 | 24.68 | 5E-07 |
| 75. | Φ (TSM + age + s-TNn + w-R10^2^ + w-R10 + age * w-R10^2^) ρ (sex) | 9 | 3767.65 | 24.70 | 5E-07 |
| 76. | Φ (TSM + s-TNn + w-TNn + w-R10^2^ + s-R10 + w-R10) ρ (sex) | 9 | 3767.68 | 24.73 | 5E-07 |
| 77. | Φ (TSM + age + s-TNn + w-TNn + w-R10^2^ + w-R10 + age * w-R10^2^) ρ (sex) | 10 | 3767.96 | 25.00 | 5E-07 |
| 78. | Φ (TSM + age + w-TNn + w-R10^2^ + s-R10 + w-R10 + age * w-R10^2^) ρ (sex) | 10 | 3768.09 | 25.13 | 4E-07 |
| 79. | Φ (TSM + age + s-TNn + w-TNn + w-R10^2^ + s-R10 + w-R10 + age * s-R10) ρ (sex) | 11 | 3768.39 | 25.43 | 4E-07 |
| 80. | Φ (TSM + age + w-TNn + w-R10^2^ + s-R10 + w-R10 + age * w-R10^2^ + age * s-R10) ρ (sex) | 11 | 3768.56 | 25.60 | 3E-07 |
| 81. | Φ (TSM + age + s-TNn + w-R10^2^ + s-R10 + w-R10 + age * w-R10^2^) ρ (sex) | 10 | 3768.63 | 25.67 | 3E-07 |
| 82. | Φ (TSM + age + s-TNn + w-R10^2^ + s-R10 + w-R10 + age * w-R10^2^ + age * s-R10) ρ (sex) | 11 | 3769.12 | 26.16 | 3E-07 |
| 83. | Φ (TSM + age + s-TNn + w-TNn + w-R10^2^ + s-R10 + w-R10 + age * w-R10^2^) ρ (sex) | 11 | 3769.62 | 26.67 | 2E-07 |
| 84. | Φ (TSM + age + s-TNn + w-TNn + w-R10^2^ + s-R10 + w-R10 + age * w-R10^2^ + age * s-R10) ρ (sex) | 12 | 3770.06 | 27.10 | 2E-07 |
| 85. | Φ (TSM + s-TNn + w-TNn) ρ (sex) | 6 | 3772.91 | 29.96 | 4E-08 |
| 86. | Φ (TSM + age + s-TNn + w-TNn) ρ (sex) | 7 | 3773.04 | 30.09 | 4E-08 |
| 87. | Φ (TSM + s-TNn + w-TNn + s-R10) ρ (sex) | 7 | 3774.63 | 31.67 | 2E-08 |
| 88. | Φ (TSM + age + s-TNn + w-TNn + s-R10) ρ (sex) | 8 | 3774.76 | 31.81 | 2E-08 |
| 89. | Φ (TSM + age + s-TNn + w-TNn + s-R10 + age * s-R10) ρ (sex) | 9 | 3775.60 | 32.64 | 1E-08 |
| 90. | Φ (TSM + s-TNn) ρ (sex) | 5 | 3778.22 | 35.27 | 3E-09 |
| 91. | Φ (TSM + age + s-TNn) ρ (sex) | 6 | 3778.47 | 35.51 | 2E-09 |
| 92. | Φ (TSM + w-TNn) ρ (sex) | 5 | 3778.89 | 35.93 | 2E-09 |
| 93. | Φ (TSM + age + w-TNn) ρ (sex) | 6 | 3779.11 | 36.15 | 2E-09 |
| 94. | Φ (TSM + w-TNn + s-R10) ρ (sex) | 6 | 3779.67 | 36.71 | 1E-09 |
| 95. | Φ (TSM + age + w-TNn + s-R10) ρ (sex) | 7 | 3779.89 | 36.93 | 1E-09 |
| 96. | Φ (TSM + s-TNn + s-R10) ρ (sex) | 6 | 3780.19 | 37.24 | 1E-09 |
| 97. | Φ (TSM + age + s-TNn + s-R10) ρ (sex) | 7 | 3780.44 | 37.48 | 9E-10 |
| 98. | Φ (TSM + age + w-TNn + s-R10 + age * s-R10) ρ (sex) | 8 | 3780.86 | 37.90 | 7E-10 |
| 99. | Φ (TSM + age + s-TNn + s-R10 + age * s-R10) ρ (sex) | 8 | 3781.27 | 38.31 | 6E-10 |
| 100. | Φ (TSM) ρ (sex) | 4 | 3781.81 | 38.86 | 4E-10 |
| 101. | Φ (TSM + age) ρ (sex) | 5 | 3782.12 | 39.16 | 4E-10 |
| 102. | Φ (TSM + s-R10) ρ (sex) | 5 | 3783.66 | 40.70 | 2E-10 |
| 103. | Φ (TSM + age + s-R10) ρ (sex) | 6 | 3783.96 | 41.01 | 2E-10 |
| 104. | Φ (TSM + age + s-R10 + age * s-R10) ρ (sex) | 7 | 3784.98 | 42.02 | 9E-11 |

**Table S2.** Linear mixed models ranked by AIC predicting initiation date of laying in kestrels, based on 2,411 breeding attempts recorded in Western Finland, with degrees of freedom (*df*) and model weights displayed. See Table S1 for other abbreviation definitions.

| # | **voles** | **w-R10** | **w-TNn** | **w-R10**  **× voles** | **w-TNn**  **× voles** | ***df*** | **AIC** | **ΔAIC** | **Weight** |
| --- | --- | --- | --- | --- | --- | --- | --- | --- | --- |
| 1. | × | × |  | × |  | 7 | 17703.71 | 0.00 | 0.41 |
| 2. | × | × | × | × |  | 8 | 17704.92 | 1.20 | 0.22 |
| 3. | × | × |  |  |  | 6 | 17706.20 | 2.48 | 0.12 |
| 4. | × | × | × | × | × | 9 | 17706.77 | 3.06 | 0.09 |
| 5. | × | × | × |  |  | 7 | 17707.72 | 4.00 | 0.05 |
| 6. | × |  |  |  |  | 5 | 17707.92 | 4.21 | 0.05 |
| 7. | × |  | × |  |  | 6 | 17709.21 | 5.50 | 0.03 |
| 8. | × | × | × |  | × | 8 | 17709.49 | 5.78 | 0.02 |
| 9. | × |  | × |  | × | 7 | 17710.98 | 7.26 | 0.01 |
